# Long non-coding RNAs expression and regulation across different brain regions in primates

**DOI:** 10.1038/s41597-024-03380-3

**Published:** 2024-05-28

**Authors:** Mohit Navandar, Constance Vennin, Beat Lutz, Susanne Gerber

**Affiliations:** 1 Institute for Human Genetics, University Medical Center of the Johannes Gutenberg University Mainz, Mainz, Germany; 2 Leibniz Institute for Resilience Research, 55122 Mainz, Germany; 3 Institute of Physiological Chemistry, University Medical Center of the Johannes Gutenberg University Mainz, 55128 Mainz, Germany

**Keywords:** Transcriptomics, Data mining

## Abstract

Human and non-human primates have strikingly similar genomes, but they strongly differ in many brain-based processes (e.g., behaviour and cognition). While the functions of protein-coding genes have been extensively studied, rather little is known about the role of non-coding RNAs such as long non-coding RNAs (lncRNAs). Here, we predicted lncRNAs and analysed their expression pattern across different brain regions of human and non-human primates (chimpanzee, gorilla, and gibbon). Our analysis identified shared orthologous and non-orthologous lncRNAs, showing striking differences in the genomic features. Differential expression analysis of the shared orthologous lncRNAs from humans and chimpanzees revealed distinct expression patterns in subcortical regions (striatum, hippocampus) and neocortical areas while retaining a homogeneous expression in the cerebellum. Co-expression analysis of lncRNAs and protein-coding genes revealed massive proportions of co-expressed pairs in neocortical regions of humans compared to chimpanzees. Network analysis of co-expressed pairs revealed the distinctive role of the hub-acting orthologous lncRNAs in a region- and species-specific manner. Overall, our study provides novel insight into lncRNA driven gene regulatory landscape, neural regulation, brain evolution, and constitutes a resource for primate’s brain lncRNAs.

## Introduction

In the course of evolution, the human brain has evolved into a most complex organ. It differs in several aspects from the brain of non-human primates in terms of its development, maturation, size, and functional complexity, despite having a genome similarity e.g., of ~98.5% with a chimpanzee^1–4^. Transcriptional regulation plays a fundamental role in brain architecture and functionality. Previous studies discovered that a massive proportion of human protein-coding sequences show significant similarities with other primates. However, despite these similarities, a minor fraction of protein-coding genes are known to drive human-specific brain regulatory mechanisms^1,5–9^. In humans, non-coding RNAs contribute to around 98% of total transcriptional output; however, understanding their impact on brain transcriptional regulation and evolution has remained limited. One of the classes of non-coding RNAs, the long non-coding RNAs (lncRNAs), are larger than 200 nucleotides in length with limited protein-coding potential^10,11^. LncRNAs are known to be involved in various functions underlying developmental and disease conditions by regulating multiple processes, such as at epigenetic (e.g., by recruiting chromatin remodeling complexes or acting as a scaffold, etc.), transcriptional (e.g., by binding and recruiting transcription factor/RNA binding proteins, etc.) and post-transcriptional (e.g., by mediating RNA modifications, etc.) levels. It has been documented that one-third of lncRNAs has emerged within the primate lineage, and around 40% of them are brain-specific, which implies that lncRNAs are essential for brain development and higher cognitive abilities^12,13^.

Several studies have already reported the involvement of lncRNAs in brain function and evolution. The Metastasis Associated Lung Adenocarcinoma Transcript 1 (MALAT1), regulates genes for synapse formation by recruiting SR-family proteins to the transcriptionally active locus^14^. The primate lncRNA LncND is known to regulate Notch signaling by sequestering miRNA, thus playing a role in brain expansion^15^. LncRNAs are also known to play a role in neuropathological disorders. In the prefrontal cortex, downregulation of lncRNA LINC00473 affects mood exclusively in females leading to depression, thus regulating gene expression and physiology in a sex-specific manner^16^. In the case of schizophrenia, bipolar disorder, and neurodegenerative diseases, specific lncRNAs have been shown to be dysfunctional in several brain regions such as the Brodmann’s Area (BA) 24, BA9 and BA11, temporal gyrus, etc.^17,18^. Recent exploration of the molecular features of striatum development has led to the screening of human-specific long intergenic non-coding RNAs (lincRNAs), potentially playing a role in brain development and synaptic organization and providing insight into the evolutionary divergence of the striatum^19^. *In vitro* studies of human and non-human primate organoids revealed that structurally conserved primate lncRNAs are transiently expressed during human cortical differentiation and regulate cell type-specific genes^20^. The analysis of transcriptomes from different organs across developmental stages from several species revealed the evolutionary importance of lncRNAs and its contribution to stage-specific expression patterns^21,22^. The exploration of lincRNAs across multiple tissues of different species revealed faster evolution and strong tissue specificity of hominid-specific lncRNAs^23,24^. A study investigated the lncRNA expression in the brain of rhesus macaques across different age groups, elucidating the presence of spatial, age-dependent, and sex-specific expression of lncRNAs^25^. Xu and Li *et al*. determined the unique human transcriptome features based on the expression profiles of protein-coding genes across different brain regions of primates^5^. However, gene regulation is highly complex. Hence, despite these studies, there has been a gap toward understanding lncRNA features across multiple brain regions of different primates.

Our study analysed long non-coding RNAs (lncRNAs) in eight different brain regions across human, chimpanzee, gorilla, and gibbon, and identified shared orthologous and non-orthologous lncRNAs with distinct genomic features. Expression analysis of the orthologous lncRNAs revealed human specific lncRNAs expression in neocortical and subcortical regions, while it showed similar expression in cerebellum of both the species. We have evidence that the regulation of various biological processes, ranging from neurogenesis to synaptic functions, is driven by these lncRNAs. We also showed that orthologous lncRNAs regulate different set of genes involved in similar biological functions in a species-specific manner. Overall, the present study provides insights into the lncRNA-driven gene regulatory landscape, neural gene regulation and comprehensive catalogue of primates brain lncRNAs.

## Results

### lncRNA predictions, its orthologs, and genomic features from the human and non-human primate’s brain regions

**Fig. 1:**
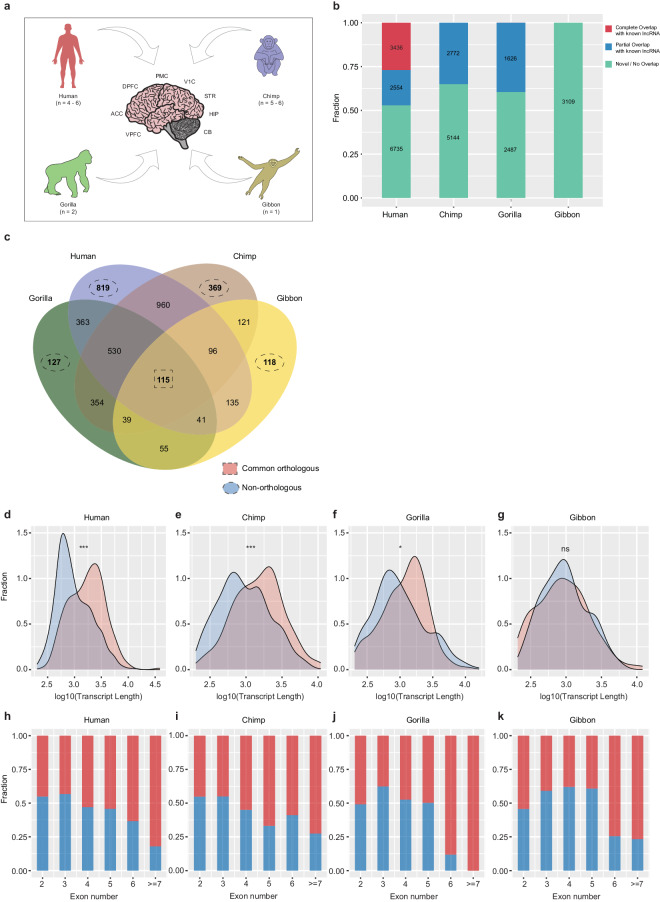
LncRNA predictions from the human and non-human primate’s brain regions, its orthologs, and genomic features. (**a**) Overview of the data used for predicting lncRNAs. (**b**) Bar plot representation of the total fraction and number of (completely overlapping with known lncRNAs, partial overlapping with known lncRNAs, novel) lncRNAs predicted in human, chimpanzee, gorilla, and gibbon. (**c**) Venn diagram depicts common orthologs and non-orthologs lncRNA groups predicted by OrthoMCL amongst human, chimpanzee, gorilla, and gibbon. (**d**–**g**) Density plot shows the differences between the transcript length of common orthologous (light red) and non-orthologous (light blue) lncRNAs from human, chimpanzee, gorilla, and gibbon [***p-value < 0.001, **p-value < 0.01]. (**h**–**k**) Bar plot represents the fraction of transcripts with the number of exons between the common orthologous (red) and non-orthologous (blue) lncRNAs from all four species.

We extracted publicly available datasets of human and non-human primates such as chimpanzees, gorillas, and gibbons with eight different brain regions, including non-cortical region such as cerebellum (CB), sub-cortical regions such as striatum (STR), hippocampus (HIP), and the five neocortical regions such as anterior cingulate cortex (ACC), dorsolateral prefrontal cortex (DPFC), ventromedial prefrontal cortex (VPFC), premotor cortex (PMC), primary visual cortex (V1C) (Fig. 1a)^5^. After applying the lncRNA prediction pipeline (Method section), we could predict 12725, 7916, 4113, and 3109 lncRNA transcripts with different genomic classes from brain areas of humans, chimpanzees, gorillas, and gibbons, respectively (Fig. 1b, Supplement Fig. 1a). In the human samples, approximately 27% out of the 12,725 lncRNAs completely overlapped with already documented lncRNAs from GENCODE, 20% partially overlapped with the known lncRNAs, and 53% of the lncRNAs were novel. In non-human primates, we observed a partial overlap of approximately 35% and 40% with the known lncRNAs from chimpanzees and gorillas, respectively. Around 65%, 60%, and all predicted lncRNAs were novel in chimpanzees, gorillas, and gibbons, respectively (Fig. 1b). Our orthologous analysis revealed the lncRNA’s association with orthologous or non-orthologous classes, each with several different groups (Fig. 1c, figshare^26^: Orthologous_lncRNA_information.xlsx). We observed 115 orthologous groups of lncRNAs, known as shared orthologs, across human and non-human primates. At the same time, 819, 369, 127, and 118 groups were non-orthologous in humans, chimpanzees, gorillas, and the gibbon, respectively (Fig. 1c). Upon performing genomic feature analysis of shared orthologous and non-orthologous lncRNAs, we observed significant differences in transcript length and fraction of exons per transcript (Fig. 1 d:g and h:k). The transcript length of shared orthologous lncRNAs was significantly higher than non-orthologous lncRNAs in humans, chimpanzees, and gorillas but not in gibbon (probably due to low sample size). Shared orthologous lncRNA transcripts showed a higher fraction of transcripts containing greater number of exons compared to non-orthologous transcripts. These observations suggest that orthologous lncRNAs have evolutionary conservation, preferences for splice site, and ability to accommodate the RNA regulatory complexes to assist the interactions with other nucleic acid complexes and proteins^27^.

### The expression pattern of lncRNAs differ between cortical and non-cortical regions

**Fig. 2:**
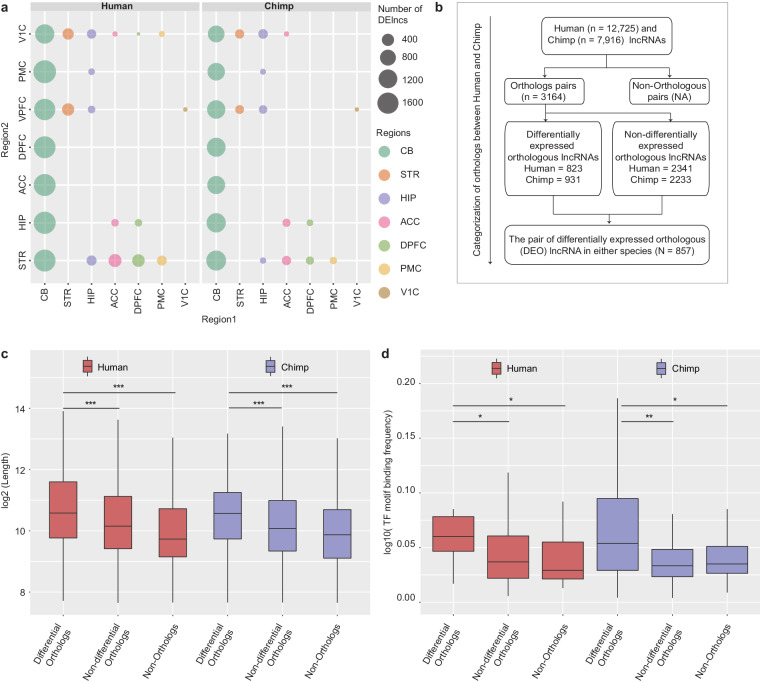
Classification of lncRNAs based on expression and orthologs. (**a**) Summary of differential expression analysis of lncRNAs across the brain regions of human and chimpanzee. (**b**) Classification of lncRNAs from human and chimpanzee based on the orthologous/non-orthologous status and differential expression in any of the brain region of either species. The last box represents the pair of differentially expressed orthologous (DEO) lncRNAs in either species and pair count (N = 857). (**c**) Box plot shows the differences in transcript length amongst the differential orthologs, non-differential orthologs, and non-orthologous lncRNAs from human and chimpanzee [***p-value < 0.001]. (**d**) Box plot represents the transcription factor (TF) motif binding frequency between differential orthologs, non-differential orthologs, and non-orthologous lncRNAs from human and chimpanzee [**P-value < 0.01, *P-value < 0.05].

Differential expression analysis was performed to understand the expression pattern of lncRNAs in different brain regions. To retain the statistical power during analysis, we considered only human (n = 5-6) and chimpanzee (n = 4-5) (due to a higher number of samples) (Fig. 1a). We observed that the number of differentially expressed (DE) lncRNAs were highly similar in both species (Fig. 2a). Interestingly, this analysis showed that a greater number of DE lncRNAs in CB compared with the rest of the brain regions (Fig. 2a). In both species, within neocortical regions (ACC, DPFC, VPFC, PMC, and V1C) and sub-cortical (HIP and STR) regions number of DE lncRNAs were less compared to CB (Fig. 2a). These observations indicate that transcriptional patterns of lncRNAs differ significantly between the cortical and non-cortical (CB) brain regions, but overall brain lncRNAs transcriptome maintained homogeneity between human and chimpanzee.

### Differentially expressed orthologous lncRNAs have distinct genomic features

To elucidate features of DE lncRNAs from the brain regions of these species, we decided to classify lncRNAs as per differential expression and ortholog status (orthologous vs. non-orthologous) (Fig. 2b). Our classification resulted in 3164 orthologous pairs of lncRNAs between human and chimpanzee. Further, orthologous lncRNAs were categorized into differentially and non-differentially expressed lncRNAs (Fig. 2b). In human, 823 DE lncRNAs were orthologous to 931 chimpanzee DE lncRNAs, while 2341 human lncRNAs were orthologous to 2233 lncRNAs in chimpanzee with non-differential expression (Fig. 2b). Upon exploring the features (such as transcript length, exon numbers, transcription factor (TF) motif binding frequency) of differentially expressed orthologs (DEO), non-differentially expressed orthologs (NDEO), and non-orthologs (NO) lncRNAs, we observed that DEO lncRNAs have significantly higher transcript length compared to NDEO and NO lncRNAs in both species (Fig. 2c). This observation indicates DEOs have inclined preferences for splicing sites, evolutionary conservation, and the ability to interact with other regulatory complexes^27^. However, a higher fraction of transcripts with a greater number of exons was observed in NDEO compared to DEO lncRNAs (Supplement Fig. 1b). Also, the TF motif binding frequency was significantly higher in DEO lncRNAs compared to NDEO and NO lncRNAs in both species, suggesting the relevance of DEO lncRNAs in the transcriptional regulation of brain region’s states (Fig. 2d). Overall, these results demonstrate the unique genomic features of DEOs and highlight their potential role in specialized functions in the brain regions.

### DEO lncRNAs from human exhibited unique expression profiles in neocortical and sub-cortical regions

**Fig. 3:**
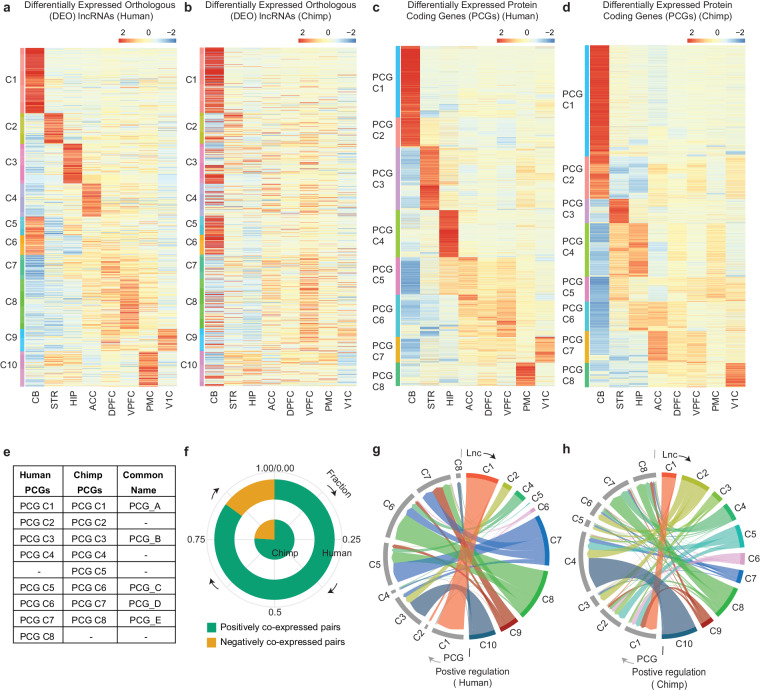
Expression pattern of DEO lncRNAs from human and chimpanzee along with its regulation. (**a**) Heatmap representing the region-specific and shared expression profile divided into 10 clusters of the DEO lncRNAs across different brain regions of the human. (**b**) Heatmap representing expression profiles of the DEO lncRNAs across different brain regions of the chimpanzee arranged according to clustering of the human orthologs. (**c**) Clusters of the differentially expressed PCGs expression across different brain regions of the human in heatmap representation. (**d**) Differentially expressed PCGs across different brain regions of the chimpanzee clustered into the 8 distinct clusters and shown in the heatmap format. (**e**) Chart represents the reannotation of the clusters from PCGs based on the similar expression profiles in both species. (**f**) Circular plot represents the total fraction of positively and negatively co-expressed lncRNA-PCG pairs in human and chimpanzee. (green: positively co-expressed pairs; gold: negatively co-expressed pairs). (**g**) Circular layout representation for the fraction of the positively co-expressed lncRNA-PCG pairs from human brain regions. (**h**) Circular layout representation for the fraction of the positively co-expressed lncRNA-PCG pairs from chimpanzee brain regions.

Based on the previous observations of the unique genomic features of DEO lncRNAs, we decided to investigate the expression profiles and gene regulatory landscape across different brain regions of these species. We extracted the pairs of orthologous lncRNAs differentially expressed (N = 857) in either species (Fig. 2b). Further, we implemented k-means clustering to determine the expression patterns amongst these lncRNAs in human. The analysis resulted in 10 distinct clusters (Fig. 3a). Chimpanzee lncRNAs were arranged in clusters as per its orthologs in human (Fig. 3b). CB region (cluster C1, C5, C6) exhibits similar expression pattern of DEO lncRNAs in both species (Fig. 3a,b; Supplement Fig. 2a). Subcortical areas such as STR (cluster C2) and HIP (cluster C3, C5, and C7) showed specific expression patterns of DEO lncRNAs in human compared to chimpanzee (Fig. 3a,b, Supplement Fig. 2a). In contrast to chimpanzee neocortex, cluster C4, C8, C9 showed human neocortex specific expression pattern (Fig. 3a,b, Supplement Fig. 2a). Further, we observed that human neocortical and subcortical areas showed similar expression profile for the DEO lncRNAs (cluster C7 and C10) (Fig. 3a,b, Supplement Fig. 2a). These observations indicate that neocortical and subcortical regions DEO lncRNAs have distinct expression patterns in human compared to chimpanzee.

To understand the impact of lncRNAs on the protein-coding genes (PCGs), we performed a differential gene expression analysis of PCGs across different brain regions of both species. Using the k-means algorithm, we observed eight different clusters of DE PCGs in both organisms (Fig. 3b,c). Similar to DEO lncRNAs, in CB region (clusters PCG C1, PCG C2) we observed similar expression profiles of DE PCGs in both species (Fig. 3b,c, Supplement Fig. 1b). Subcortical regions such as STR (cluster PCG C3) and HIP (cluster PCG C4) exhibit similar expression patterns in both species (Fig. 3b,c, Supplement Fig. 2b). Human neocortical region-specific clusters (PCG C6 and PCG C7) showed a similar expression pattern with the chimpanzee neocortical region-specific clusters (PCG_C7 and PCG C8) (Fig. 3b,c, Supplement Fig. 2b). Strikingly, we also observed that neocortical region (cluster PCG- C2 [shared with CB]) showed unique expression pattern in chimpanzee compared to human (Fig. 3b,c, Supplement Fig. 2b). These observations indicate that the clusters of PCGs showed high similarity of expressions amongst both species. Further to make the annotation consistent, we reannotated clusters as per the expression profiles, (as shown in Fig. 3e).

### DEO lncRNAs and PCGs exhibited a higher fraction of positive co-expression in both species

Next, we aimed to implement co-expression analysis of lncRNAs and PCGs to determine the potential gene regulatory landscape, as co-expression patterns enable to infer shared regulatory features and functional biological processes^28^. To evaluate co-expression, we applied Pearson’s correlation coefficient calculation (Method section and figshare^26^: Co-expressed_pairs_lncRNA-PCG.xlsx). During our analysis, we observed a high fraction of positively co-expressed pairs compared to negatively co-expressed pairs in both species (Fig. 3f). Hence, we addressed the positively co-expressed pairs of lncRNAs-PCGs for downstream analysis.

In human CB, we observed distinct co-expression of CB specific lncRNAs (Lnc: C1) and PCGs (PCG: C1, C2) (Fig. 3g), however in chimpanzee CB related PCGs were co-expressed with the lncRNAs from multiple clusters (Fig. 3h). In sub-cortical areas, PCGs related to STR region (PCG C3) of both the species, were co-expressed majorly with the human STR specific lncRNAs (Lnc:C2) and sub, neocortical shared lncRNAs (Lnc: C10) (Fig. 3h) and chimpanzee lncRNA (Lnc: C2) (Fig. 3g). Contrary to our expectations, no co-expression pairs were found between lncRNAs and PCGs in human HIP region, however positively co-expressed lncRNA-PCG pairs were found in chimpanzee in the same region. Interestingly in neocortex, we observed a higher fraction for the number of co-expressed pairs of lncRNA and PCG in human compared to chimpanzee.

### Positively co-expressed lncRNAs with PCGs regulate diverse neural functions in region specific manner

**Fig. 4:**
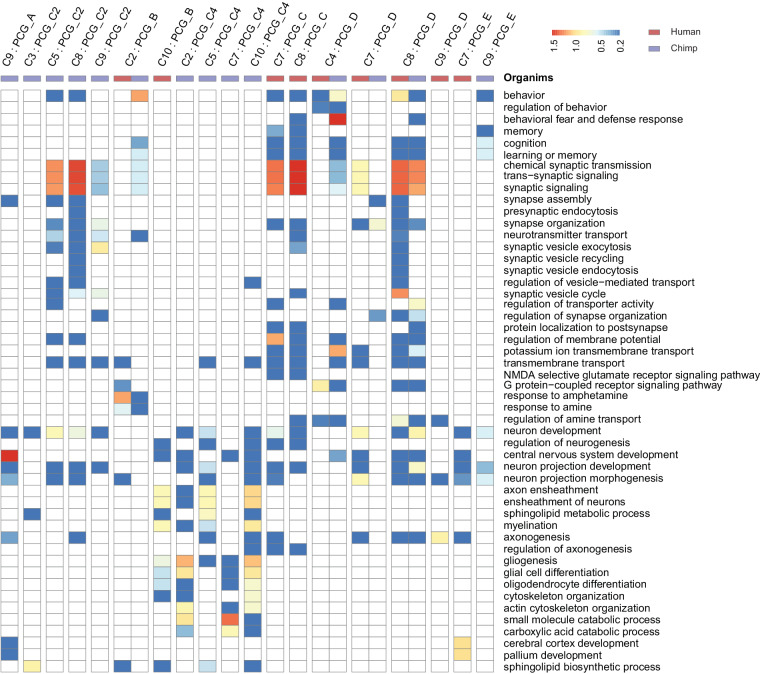
Gene ontologies of positively co-expressed lncRNAs with PCGs. Heatmap of the GO ontologies for the PCGs positively co-expressed with lncRNA-PCGs clusters. (red: human; violet: chimpanzee). Each column of the heatmap represents the lncRNA-PCGs pairs from specific clusters and the row represents the PCGs enriched for a particular biological process.

To evaluate the functional enrichment of respective PCGs from the lncRNA-PCG co-expression pairs of clusters, we implemented the gene ontology (GO) analysis (Method section and Fig. 4). In chimpanzee CB region (C9: PCG_A) we observed enrichment of the central nervous system (CNS) development genes, but we did not observe any GO enrichment in CB (Fig. 4). Since chimpanzee PCG_C2 showed unique expression pattern in neocortical regions unlike human, we observed GO enrichments such as chemical synaptic transmission, trans-synaptic signaling, assembly, neurotransmitter transport, synaptic vesicle recycling, synaptic vesicle exo/endocytosis associated with PCGs co-expressed with different lncRNA clusters (C5, C8, and C9).

In one of the sub-cortical regions i.e., STR, we observed functional enrichment of response to amine as a common ontology in both species (C2: PCG_B), but chemical synaptic transmission, synaptic signaling, neurotransmitter transport related genes were specifically co-expressed in chimpanzee STR. However, we also observed functional enrichment of biological processes such as the regulation of neurogenesis, CNS development, axon ensheathment, myelination and gliogenesis uniquely in human STR (C10: PCG_B). In the region of chimpanzee HIP, we observed GO terms such as the regulation of neurogenesis, CNS development, axon ensheathment, myelination, axonogenesis, gliogenesis, and cytoskeleton organization were enriched in most of the lncRNA-PCG pair sets.

In neocortex, co-expressed PCGs were enriched for biological processes such as chemical synaptic transmission, synaptic signaling, synapse organization, ion transport, transmembrane transport, learning and memory, CNS development in both the species (C8: PCG_D). However, functional ontologies such as synaptic vesicle exo/endo-cytosis, neurotransmitter transport, synaptic vesicle cycle processes were specifically enriched in human neocortex (C8: PCG_D). Overall, our observations suggest that lncRNAs are essential players in regulating diverse neurobiological processes in specific brain regions.

### Regulation of neural functions by hub lncRNAs in a species-specific manner

**Fig. 5:**
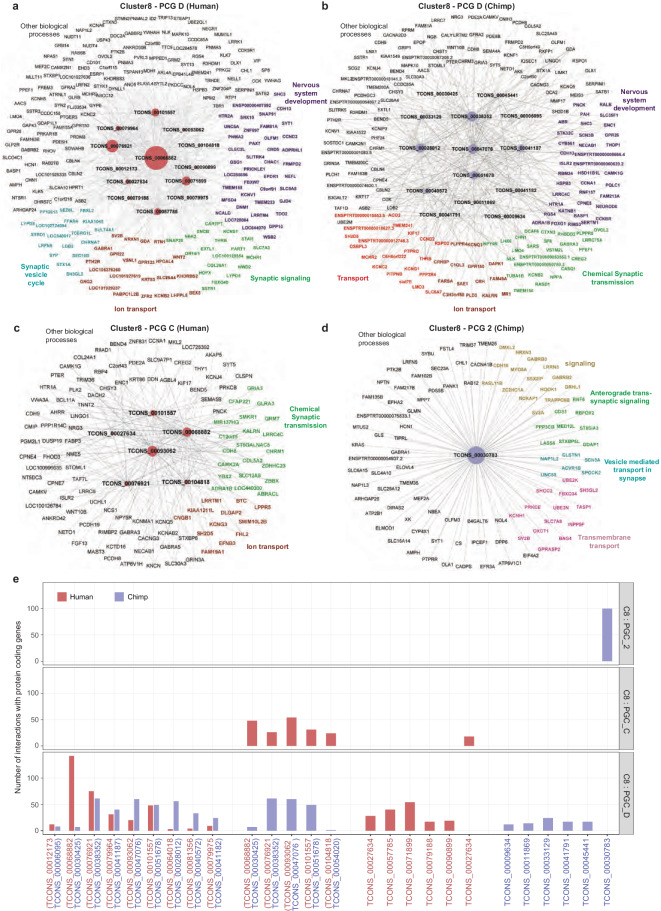
Regulation of neural functions by hub lncRNAs. (**a**) Network plot representing the co-expression network of C8 - PCG D network from human. Genes regulating the specialized biological processes are labeled in different color notations. The size of the hub node of lncRNAs indicates the number of co-expressed PCGs. (**b**) Network plot representing the co-expression network of C8 - PCG D network from chimpanzee. Genes regulating the specialized biological processes are labeled in different color notations. The size of the hub node of lncRNAs indicates the number of co-expressed PCGs. (**c**) Network plot representing the co-expression network of C8 - PCG C network from human. Genes regulating the specialized biological processes are labeled in different color notations. The size of the hub node of lncRNAs indicates the number of co-expressed PCGs. (**d**) Network plot representing the co-expression network of C8 - PCG C2 network from chimpanzee. Genes regulating the specialized biological processes are labelled in different color notations. The size of the hub node of lncRNAs indicates the number of co-expressed PCGs. (**e**) Bar plot depicting an overview of the number of interactions of hub lncRNAs and its orthologous hub lncRNAs in both species and the above-mentioned networks.

Next, we aimed to implement the co-expression network analysis to determine the potential gene regulatory landscape with hub lncRNA(s) (figshare^26^: Hub_lncRNAs_co-expression _of_PCGs.xlsx). Since the neocortex is a recently evolved structure in the brain responsible for unique capabilities of humans compared to other mammals^29,30^ and from our previous observations of gene expression profiles and GO analysis, we decided to investigate the gene regulatory network from neocortex-specific clusters. In neocortex, network analysis of co-expressed pair from C8: PCG_D revealed that massive numbers of co-expressed PCGs in both species (Fig. 3g,h). We identified the hub acting lncRNAs from both the networks, human (C8: PCG_D; n = 13) and chimpanzee (C8: PCG_D; n = 14) and observed a common and unique set of co-expressed PCGs from both networks (Supplement Fig. 3a and figshare^26^: Common_and_unique_PCGs_C8_PCG_D.xlsx). LncRNAs were co-expressed with common genes such as CBNL2, LMO4, GDA, DLX1, CCND2, SERPINI1, OLFM1, CCND2, etc. in both species. We observed human network-specific synaptic genes such as AMPH, MEF2C, STXBP1, SNAP25, SYN1/2, SYP, and SYT1, while CHRM1/3, GRIP1, KCNQ5, KCNF1, NETO1, KCNJ4 genes were specific to chimpanzee network (Supplement Fig. 3a and figshare^26^: Common_and_unique_PCGs_C8_PCG_D.xlsx). In these networks from both the species, co-expressed PCGs were involved in biological processes such as synaptic signaling, ion transport, nervous system development (Fig. 5a,b, figshare^26^: Common_and_unique_PCGs_C8_PCG_D.xlsx), however synaptic vesicle cycle related PCGs were co-expressed specifically in human network (Fig. 5a, figshare^26^: Common_and_unique_PCGs_C8_PCG_D.xlsx). In human neocortical network C8: PCG_D, one of the hub lncRNAs ‘TCONS_00068882’ has a greater number of co-expressed genes. However, its orthologous lncRNA ‘TCONS_00030425’ in chimpanzee was co-expressed with a smaller number of PCGs genes (Fig. 5a,b,e). In chimpanzee network C8: PCG_D, hub lncRNAs such as ‘TCONS_00038352’ and ‘TCONS_00047076’ were co-expressed with a similar, but higher number of PCGs compared to other hub lncRNAs from the same network (Fig. 5b,e). However, its orthologs were not co-expressed with PCGs in the same neocortical network from human (C8: PCG_D), but with shared cluster of neocortical and sub-cortical region from human (C8: PCG_C) (Fig. 5a,b,e).

Due to the unique GO from neocortical specific cluster C8: PCG_C in humans (Fig. 4a), we also decided to investigate the co-expression network of lncRNA-PCGs, resulted in PCGs regulating chemical synaptic transmission-related genes such as GABRA5, GRIA3, GRIN3A, KCNQ5, etc. and ion transport genes like KCNG3, CNCG1, LPPR5. In this network, hub lncRNAs (n = 5) such as ‘TCONS_00068882’ and ‘TCONS_00093062’ were co-expressed with a similar number of PCGs (Fig. 5c,e). Interestingly, our previous observation showed that C8 lncRNAs of chimpanzee (having expression in CB, contrast to human) were co-expressed with the PCG_C2. Its co-expressed network genes were related to synaptic signaling, vesicle-mediated transport, transmembrane transport, and other critical biological processes with hub lncRNA as “TCONS_00030783” (Fig. 5d,e).

From the 54 predicted human hub lncRNAs, the expression of 20 hub lncRNAs were analyzed using *in vitro* model of human neuronal differentiation (Method and Supplement Fig. 3). Among the 20 lncRNAs analyzed, 14 showed either no enrichment compared with neuronal progenitor cells (NPC) (e.g., TCONS_00096884, TCONS_00002162) or small enrichment (fold change < 5 compared with NPC) (TCONS_00002593), whereas 4 lncRNAs showed high enrichment (fold change up to 35 compared with NPC) during the neuronal differentiation (Supplement Fig. 3c,d). A constant increased expression of the majority of the lncRNAs during neuronal differentiation and maturation were detected (TCONS_00002593, TCONS_00088899) (Supplement Fig. 3c,d). Few of lncRNAs (e.g., TCONS_00002301, TCONS_00076921) showed a peak of expression in the intermediate progenitors cells and a decreased expression in mature neurons (Supplement Fig. 3c,d). Surprisingly, the TCONS_00079964 could not be detected in the *in vitro* model used. The distinctive expression patterns of lncRNAs were observed during neuronal differentiation suggest its role in regulating a specific set of genes essential for the processes of neuronal differentiation and/or maturation (Supplement Fig. 3c,d). Overall, our observations suggest that hub lncRNAs regulate different sets of genes having similar, but important neural functions such as synapse regulation in a species-specific manner.

## Discussion

The emergence of understanding the complex molecular regulation of brain regions and its evolution across primates has been one of the hot topics in evolutionary biology. Diverse types of RNAs, transcription factors, and epigenetic factors play an extraordinary role in regulating and evolving this intricate molecular architecture. In this study, we investigated one of the types of non-coding RNAs, the lncRNAs, across eight different brain regions of primates including human, chimpanzee, gorilla, and gibbon (Fig. 1a). Our predicted novel and already known lncRNA transcripts are a new resource and can contribute to understanding of the lncRNA driven molecular regulation of brain regions and their evolution.

Brain specific lncRNAs show higher sequence conservation than the lncRNA expressed in other tissues^20,31,32^. To understand the conservation, we performed orthology analysis. Orthology analysis revealed orthologous lncRNAs common amongst all species and non-orthologous lncRNAs. LncRNAs are versatile in their mode of action, hence the level of conservation could potentially specify the most probable function of lncRNAs^33,34^. However, ample of non-conserved lncRNAs play a critical role in diverse biological processes. Shared orthologous lncRNAs showed indications (e.g., higher transcript length, higher fraction for transcripts with greater number of exons) of functional enrichments (Fig. 1d,k). Genomic feature analysis of the DEO lncRNAs between human-chimpanzee showed a unique pattern for the genomic features such as transcript length, transcription factor binding frequency, and exon numbers per transcript. These features indicate functional enrichment for various molecular functions since it shows sign of preferences for splice site, evolutionary conservation, and ability to accommodate the RNA regulatory complexes to assist the interactions with other nucleic acid complexes and proteins^21,27^.

Upon investing the DEO lncRNAs in human and non-human primate (chimpanzee), we observed several clusters of lncRNAs and PCGs, showing region specific and/or shared expression patterns (Fig. 3a,b; Supplement Fig. 2a). These observations were consistent with the previously investigated spatiotemporal expression pattern of lncRNAs and PCGs in distinct brain regions of humans and other primates^5,25,35,36^. Co-expressed PCGs are known to play a critical role in the brain architecture of species by contributing to common and unique molecular processes^5–7^. LncRNAs are known to interact with DNA, RNA, and proteins, which impacts the regulation of neighboring (cis) and distant genes (trans) at epigenomic and transcriptomic levels, and eventually, its functions^37^. Hence, coupling the co-expression analysis would provide the details about the brain region-specific expressions and functions of lncRNAs and its co-expressed PCG^25,28^. For example, one of the evolutionarily conserved lncRNAs, TUNA (TCL1 Upstream Neuron-Associated lincRNA or megamind) is essential for transcriptional activation of pluripotency factors such as Nanog, Sox2, and Fgf4, which regulate pluripotency and neural differentiation of mouse ES cells^38^. In our study, we observed the impact of co-expression between lncRNAs and PCGs, resulting in a massive proportion of positive co-expression pairs in both species (Fig. 3f)^12^. We identified one of the hub lncRNAs, TCONS_00093062 ( = DLX6-AS1), in human neocortical areas (C8) being co-expressed with the genes DLX1, DLX2, DLX5, among others. Previously it has been reported that DLX6-AS1 regulates the methylation of enhancers by modulating the orchestra of activators such as DLX1/DLX2 and repressor MECP2^39^. Hence in our view, DLX6-AS1 and other lncRNAs potentially governing epigenetic profiling to maintain the architecture of the neocortical regions. The expression and function of detected hub lncRNAs such as TCONS_00002162 ( = LINC01140)^40^, TCONS_00060398 ( = SIX3-AS1)^41^, TCONS_00021941 ( = LINC02389)^42^, etc. from human brain areas were previously known in disease conditions such as cancer and also known GWAS locus for addiction associate traits. However, functional insights into these lncRNAs in the brain’s complex architecture have remained elusive.

Further, we deciphered diverse neural processes regulated by co-expressed PCGs of lncRNA–PCG pairs of different clusters combinations using GO analysis. Our analysis indicates that hippocampus and striatum related lncRNAs were part of the regulation related to axon ensheathment, myelination, axonogenesis, gliogenesis, and regulation of neurogenesis (Fig. 5). The hippocampus and striatum are both the sites for adult neurogenesis^43,44^. Overall, we observed no co-expression of lncRNA-PCGs for the human hippocampal region. This is probably due to the aging of the human donor’s brain, leading to age dependent decline in total hippocampus volume in contrast to chimpanzees and monkeys^45^.

Neural plasticity in the neocortex plays an essential role in learning and memory by performing diverse motor, sensory and cognitive tasks^46^. We observed a massive number of co-expressed pairs of lncRNA-PCGs in the human’s neocortex compared to the chimpanzee. Interestingly PCGs from these pairs in both species known to regulate biological processes such as trans-synaptic signaling, chemical synaptic transmission, synapse organization, ion transport, neuron projection development, and morphogenesis-related biological processes, which play an essential role in neural plasticity. Surprisingly, human neocortex-specific co-expressed PCGs from lncRNA-PCG network were enriched for synaptic vesicle cycle, neurotransmitter transport-related biological processes GO analysis. These results explain the lncRNA-driven synaptic network enriched in the human neocortex. PCGs from the PCG_C2 cluster of chimpanzee have shared expression patterns between CB and neocortical areas in contrast to humans. Co-expressed PCGs of network cluster C8 - PCG_C2 from chimpanzee showed the enrichment for chemical synaptic transmission, trans-synaptic signaling, vesicle-mediated transport in the synapse, transmembrane transport, etc. critical biological processes might be the result of the functional interconnectivity between CB and neocortex^47^.

During network analysis, we observed a group of novel and known hub acting lncRNAs regulating the diverse neural processes in the brain region in a species-specific manner. In the neocortex of both species, hub lncRNAs were co-expressed with genes regulating synapse related processes. These observations align with the previously reported co-expression of lncRNAs (ex. MIAT) with synapse-regulating genes^25^. However, hub lncRNAs from the neocortex (C8-PCG_D) network of human, unlike chimpanzee, specifically regulate the synaptic vesicle-related genes such as STX1A and SNAP25 (are part of SNARE-associated complex) essential for vesicular neurotransmitter release and its deregulation leads to neurological dysfunctions^48^. For example, MALAT1 lncRNA promotes synapse formation in neurons by regulating synaptogenesis-related genes^14^. Our analysis also revealed that a considerable fraction of PCGs was co-expressed within C8-PCG_D network specific to either species (Supplement Fig. 3a), suggesting that synapse regulation was orchestrated precisely. The precise regulation of the synapse related processes is known during development. Cortical synaptogenesis is delayed during development in the pre-frontal cortex and extended over five years in humans compared to a few months in chimpanzee and macaque^1^. Moreover, despite of lncRNAs’ expression is tissue and cell type specific^49^, our qPCR analysis could confirm expression of few hub lncRNAs from human during *in vitro* model of human neuronal differentiation. This analysis suggests the potential role of hub lncRNAs in regulating a set of specific genes essential for neuronal differentiation and maturation in human. Besides, the studies acknowledge that certain lncRNA knockdowns have not led to observable phenotype changes^50–52^. However, it’s crucial to clarify that this does not imply these are non-functional junk RNAs^53^. Junk RNAs serve as the foundation for the evolution of diverse lncRNAs through non-adaptive mechanisms^53^.

In conclusion, our data provides lncRNA expression profiles explaining scenarios about the complex molecular architecture of brain regions across primates. The level of conservation of lncRNAs can potentially specify the most probable function^33,34^. Given this, our orthology analysis provides a detailed overview of the orthologous status of lncRNAs across the species. Our data suggests a possible molecular architecture of lncRNA driven gene regulation in the brain, as demonstrated by the specific and shared expression patterns of lncRNAs and their associated PCGs in regions such as the neocortex in both human and chimpanzee. However, we would like to point out that our study has limitations regarding the functional aspects of lncRNA-driven regulation of molecular architecture of the brain regions due to a lack of experiments for functional studies. LncRNA can regulate the expression of PCGs either positively or negatively^54^. In the current study, we focused on the positively co-expressed lncRNAs, leaving the negatively co-expressed lncRNA part unexplored. Further research using newly developed technologies such as long-read RNA-seq, nanopore technology, and single-cell technology would be essential to filter annotation rigorously and understand the diversity and role of cell-type specific lncRNAs across different brain regions of primates.

## Methods

### Data collection

We extracted a publicly available dataset from NCBI-GEO of accession number: GSE100796^55^. For details of sample information, we referred to Xu and Li *et al*.^5^. (source data: Xu, C, Li, Q. and Efimova, O. *et al*. GEO. https://identifiers.org/geo/GSE100796 (2018)).

### Data processing

We obtained raw data in fastq format, subjected to quality check using FASTQ version v0.11.9.

- Alignment: Samples qualifying the data quality were further aligned using STAR aligner version 2.7.1a^56^.
  Command and parameters for STAR:
  STAR --runThreadN 20 --readFilesCommand zcat --outSAMtype BAM SortedByCoordinate --outSAMstrandField intronMotif --outSAMattributes NH HI NM MD AS nM jM jI XS --outFileNamePrefix <Output_File_Prefix> --genomeDir <GenomePATH> --readFilesIn <READ1> <READ2>
  GTF file preparation: To collect all possible known genes and transcripts, we prepared the reference GTF file by combining known genes and transcripts from the tracks of Ensemble Genes, NCBI RefSeq, Other RefSeq, lincRNA Transcripts, GENCODE Genes V19 and UCSC Genes using USCS table browser available for species. We used the genome version of hg19 (for human), panTro4 (chimpanzee), gorGor4 (gorilla), and nomLeu3 (gibbon).
  Results of the alignment were received in BAM file format. The processed BAM file from the same brain area and species were merged to increase the coverage using samtools merge command.
- *ab-initio* transcriptome assembly:

We used merged BAM files for the *ab initio* transcriptome assembly using StringTie version 2.0.4^57^.

Command and parameters for stringTie:

stringtie <Enter bam file> -o <output/individual_files.gtf> -p 20 -f 0.50 -m 200 -a 10 -j 3 -c 0.1 -g 10 –G <Enter reference gtf file> –rf –A <output/abund_sample.tab> -C <output/cov_ref_sample.tab> -B

Output of the StringTie was processed for removing transcript with single exon and minimum expression of transcript per million (TPM) greater than one. All the assembled transcripts from different brain regions of species were merged into a GTF file representing a single transcriptome per species using *cuffmerge* from cufflinks version v2.2.1^58^.

Command and parameters: cuffmerge -o Organism_candidates <assembly_GTF_list.txt>

To annotate each transcript from the single transcriptome per species, we used *gffcompare* version v0.11.5^59^ and reference GTF file of the species.

Command and parameters: gffcompare –r <Reference_gtf> -R -M -o sample.gffcompare.out -p Trans <GTF file>

Transcripts belonging to one of the class codes such as “c”, “e”, “s” and “p” were removed as it might represent potential errors in transcriptome assembly^60^. [figshare^26^: gffcompare_class_codes_meaning.docx].

### Detection of the protein coding potential from assemble transcripts

We removed all the transcripts with class code “ = ”, “j”, “o” if a reference gene is not classified as lncRNAs. As lncRNAs are longer than 200 nucleotides^11^, from the remaining portion, we also removed the transcripts shorter than 200 nucleotides. Since, lncRNA transcripts lacks the protein-coding potential^11^, we calculated protein-coding potential using CPC2^61^, CPAT version 1.2.4^62^ and CNIT^63^ with its default parameter.

Commands and parameters for CPC2, CPAT and CNIT:

# Using CPC2:

CPC2.py -i required.fasta -o organism_CPC2.result

# Using CPAT:

make_hexamer_tab.py -c organism_cds.random.fa -n organism.ncrna.random.fa > organism_hexamer.tsv

make_logitModel.py -x organism_hexamer.tsv -c organism_cds.random.fa -n organism.random.fa -o organism

cpat.py -g required.fasta -d organism.logit.RData -x organism_hexamer.tsv -o Organism_output_cpat

# Using CNIT

python CNIT.py–file = required.fa -o organism_cnit -m ‘ve’

To proceed with CPAT we used the coding probability cut-off of 0.364 (i.e. if coding probability >  = 0.364 then the given sequence is coding sequence, otherwise non-coding). Further, we screened the transcripts which could successfully pass the criteria of non-coding potential. Next, we implemented the TransDecoder (https://github.com/TransDecoder/TransDecoder) to determine the protein coding potential of the screened transcripts. Initially, we extracted open reading frames (ORFs) using *TransDecoder.LongOrfs* with default parameters.

Command and parameters for TransDecoder:

#Prepare fasta file from the TransDecoder:

TransDecoder-TransDecoder-v5.5.0/util/gtf_genome_to_cdna_fasta.pl <enter gtf file> <reference_genome.fa> >  <required.fasta> #Prepare gff file from gtf

TransDecoder-TransDecoder-v5.5.0/util/gtf_to_alignment_gff3.pl <enter gtf file> >  <required.gff3> # blastp

blastp –query <required.fasta> -db <prot_db> -max_target_seqs. 1 -outfmt 6 -evalue 1e-5 -num_threads 4> blastp.outfmt6

# pfam search using hmmscan

hmmscan --cpu 8 --domtblout <pfam.domtblout> <path/to/Pfam-A.hmm> longest_orfs.pep

#Run the TransDecoder

TransDecoder-TransDecoder-v5.5.0/TransDecoder.LongOrfs -t required.fasta

# Integrate blast and pfam search to find the coding regions

TransDecoder-TransDecoder-v5.5.0/TransDecoder.Predict -t required.fasta --retain_pfam_hits pfam.domtblout–retain_blastp_hits blastp.outfmt6

We scanned all the ORFs of transcript for the homology to known proteins via *blastp* and pfam search. *Blastp* was used to search for homology against the known protein database with E-value < = 1E-05. Pfam search was undertaken to search the peptide for protein domain using *hmmscan* with default parameters. Lastly, we used *TransDecoder.Predict* function to predict the ORFs. Transcripts with no ORF were further screened as potential lncRNAs for the respective species (please refer to the PrimBrainLnc database).

Next, we performed genomic classification of lncRNAs for each species using FEELnc^64^ with default settings against protein-coding genes obtained by track “Ensemble Gene” from USCS genome browser.

Command and parameters for FEELnc:

FEELnc_classifier.pl -i lncRNA.gtf -a ref_annotation.GTF > lncRNA_classes.txt

From the results we classified lncRNAs based on the positions such as upstream, downstream, overlapping and orientation of the strand as sense and antisense. Divergent type of transcripts was classified as lncRNAs with upstream sense orientation with 2 kb from its assigned protein coding.

### Orthologs identification

We used reciprocal best hit between pairs of species to cluster genes into homologous families using OrthoMCL [v2.0.9] package^65^. OrthoMCL uses Markov Cluster algorithm to group potential orthologs and paralogs based on sequence similarity. It uses MySQL and MCL program (Markov cluster algorithm) to store and compute the orthologs. We designed the schema of the OrthoMCL database as mentioned in the OrthoMCL manual with configuration file parameter *percentMatchCutoff = *50 and *evalueExponentCutoff* = 1E-5. During the all-vs-all Blast step of the OrthoMCL package, we used nucleotide blast (*blastn*) to search for similarity for transcript sequences within and across species using blast package with E-value <  = 1E-05. The output of the blast was parsed and loaded into database of OrthoMCL and *orthomclPairs* program was used to compute the pairwise relationships amongst the sequences. These pair wise relationships were used for computing the homologous groups as the clusters of orthologs.

### Differential lncRNA expression analysis

Mapped reads were used for counting reads at screened lncRNAs loci using HTSeq version 0.6.1p1^66^.

Command and parameter for HTSeq:

htseq-count -s yes -i transcript_id -t exon -f bam <sample.bam> species_Pure_lncrnas.gtf

Output of the HTSeq was used to perform the differential lncRNA expression analysis using Deseq. 2 package^67^. Differential expressions were calculated for all to all brain areas per species using *relevel* function of DESeq. 2. To define a particular lncRNA as a differentially expressed we assign the fold change of 1.5-fold and adjusted p-value cutoff (parameter *alpha*) of 0.05. We normalized the gene expression counts using *counts* functions and calculated variance stabilized counts using variance stabilized transformation as a function *vst*.

### Differential protein coding gene expression analysis

We performed the counting of the read at loci of protein coding genes (Track: Ensemble Genes) screened from USCS Table browser for each species using HTSeq. HTSeq output was used to perform the differential gene expression analysis using Deseq. 2. We used “*relevel”* function to perform the differential expression analysis for “all to all” brain regions per species. Using above mentioned cut-offs, we screened the differentially expressed protein coding genes. Further we normalized the expression counts with *counts* function and derived the variance stabilized counts using *vst* function.

### K-means clustering and selection of K

We implemented k-means clustering on the expression of DEO lncRNA and PCGs using R. We experimented with the range of k values, ensuring an exploration of a reasonable spectrum that would cover potential cluster structures in the data. For each k, we calculated the total variance, which represents the sum of squared distances between data points and their assigned cluster centroids. We generated an elbow plot, depicting the relationship between k and the corresponding total variance. The optimal k was identified at the “elbow” of the plot, where the reduction in variance begins to plateau.

### Data visualization

We used ggplot2, pheatmap, circlize packages of R.

### Co-expression based on the differential lncRNAs-PCGs expression

We screened the differentially expressed lncRNAs and PCGs in any of the brain regions within species to determine the co-expression pattern. We estimated Pearson’s correlation between lncRNA - PGGs pairs using all the samples. We considered variance-stabilized counts across the samples to calculate correlations.

### Gene ontology (GO) enrichment of PCGs of each lncRNA-PCG pair clusters and network analysis

GO analysis for PCGs of each lncRNA-PCG pair clusters were performed using ToppGene suite^68^ with cut-off p-value < 0.05 and adjusted p-value < 0.05. We implemented Cytoscape (version 3.8.0)^69^ to visualize the network by importing the correlation matrix of lncRNAs-PCGs pair. Using CytoHubba^70^ plugin of Cytoscape, we identified the hub network and sub-network from the complex network. For the network analysis we defined the cut-off for one of the topological analysis methods “degree” greater than 5. LncRNA(s) with the highest number of interactions with other PCGs in the given network was(/were) defined as (a) hub lncRNA(s).

### Screening of hub lncRNAs for validation

We screened hub lncRNAs qualifying the following criteria: (i) Number of degrees between hub lncRNA and PCGs must be greater than 5 and (ii) the presence of motif sequence at the promoter region of the hub lncRNAs. Motif prediction: we defined the promoter region as -2000 and + 1000 nucleotide base from start of the first exon. We used the homer utility to predict the motifs (data not shown) using findMotifsGenome.pl^71^.

### Cell culture

The human-induced pluripotent stem cells IMR90-4 were purchased at Wicell and maintained on matrigel-coated plate with mTeSR1 medium (Stem Cells Technologies). The neuronal progenitors cells (NPC) were generated using the PSC neural induction medium (Invitrogen) and by following manufacturer’s instructions. The NPC were maintained in Advanced DMEM/F12 (Gibco)/Neurobasal medium (Gibco) supplemented with B27 without vitamin A (Gibco), glutamax (Gibco) and Penicillin/Streptomycin (Sigma). Cells were maintained at 37 °C with 5%CO2, 95% of air in humidified atmosphere.

For the neuronal differentiation, a total of 75,000 NPC were seeded on 6-well plates coated by poly-L-ornithine (15 µg/ml) (Sigma) and laminin (10 µg/ml) (Gibco) in the presence of 5 µM ROCK inhibitors (Y-27632, Merck Millipore) in NPC medium. After 24 hours, the NPC medium were replaced by neurobasal medium supplemented with B27 (Gibco), Glutamax (Gibco) and Penicillin/Streptomycin (Sigma). Every second 2 days, 2 ml of medium were added until a final volume of 8 ml per well. Half of the medium were replaced twice a week.

### RNA extraction, reverse transcription and Real-time RT-PCR

Total RNA were extracted with Trizol (Invitrogen) from the neuronal progenitors cells (NPC) and after 14 days (intermediate progenitor cells), 28 days (immature neurons) and 42 days (mature neurons) of neuronal differentiation. From the 2 µg DNase-treated RNA, 1 µg were used for the reverse transcription (High Capacity cDNA Reverse Transcription kit, Applied Biosystem). The qPCR reactions were performed with the Power SYBR Green Master Mix (Thermofisher) and analyzed with QuantStudio^TM^ 3 System. Primer details are mentioned in figshare^26^: Primers_qPCR_hub lncRNAs.xlsx.

### Database implementation

All the predicted lncRNAs along with its detailed has been placed under the database named “PrimOrthoLnc”. PrimeOrthoLnc was implemented using Django framework (https://www.djangoproject.com/). We used SQLite relational database (https://www.sqlite.org/index.html), bootstrap (https://getbootstrap.com/), CSS and HTML for back and front end. To visualize the location of the lncRNAs from respective species we integrated UCSC genome browser (http://genome.ucsc.edu). Graph visualization was implemented with Plotly (https://plotly.com/python/).

### Supplementary information

Supplementary Figures

Supplementary Files

## Data Availability

The datasets used and generated in this study have been deposited on Figshare^26^ with the detailed description of each file. Additionally for interactive access we also provided a web server of LncRNAs predicted during this study has been placed under the database: https://csg.uni-mainz.de/software/PrimBrainLnc.

## Code availability

The source code for database is available in GitHub at https://github.com/NavandarM/PrimBrainLnc.git. The commands for tools used for lncRNA prediction and scripts for its downstream analysis is available at https://github.com/NavandarM/lncRNA-expression-and-regulation-across-different-brain-regions-in-primates.git.
