# Supplementary material for: Long non-coding RNAs expression and regulation across different brain regions in primates
Source: Sci Data. 2024 May 28;11:545. doi: 10.1038/s41597-024-03380-3 (PMC11133376; doi:10.1038/s41597-024-03380-3)
Supplement: Supplementary file 1 — Supplementary Figures [file 41597_2024_3380_MOESM1_ESM.pdf]

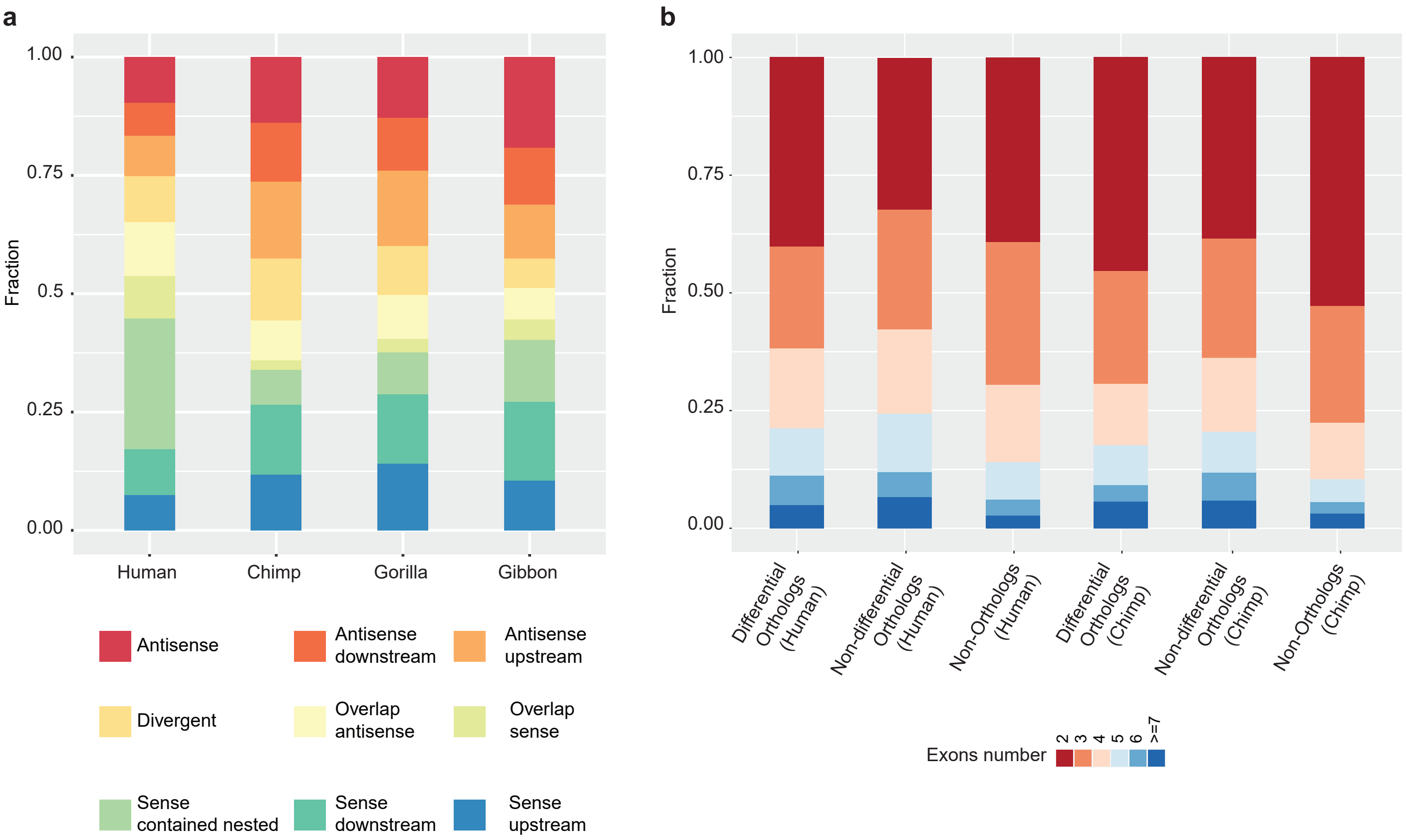

Supplement Figure 1:

(A) Bar plot representation of the fractions for lncRNA genomic classes in human, chimpanzee, gorilla, and gibbon. Each class of the transcript is annotated in different colors.

(B) Bar plot represents the distribution for fractions of exon number per transcript amongst differential orthologs, non-differential orthologs, and non-orthologous lncRNAs from human and chimpanzee.

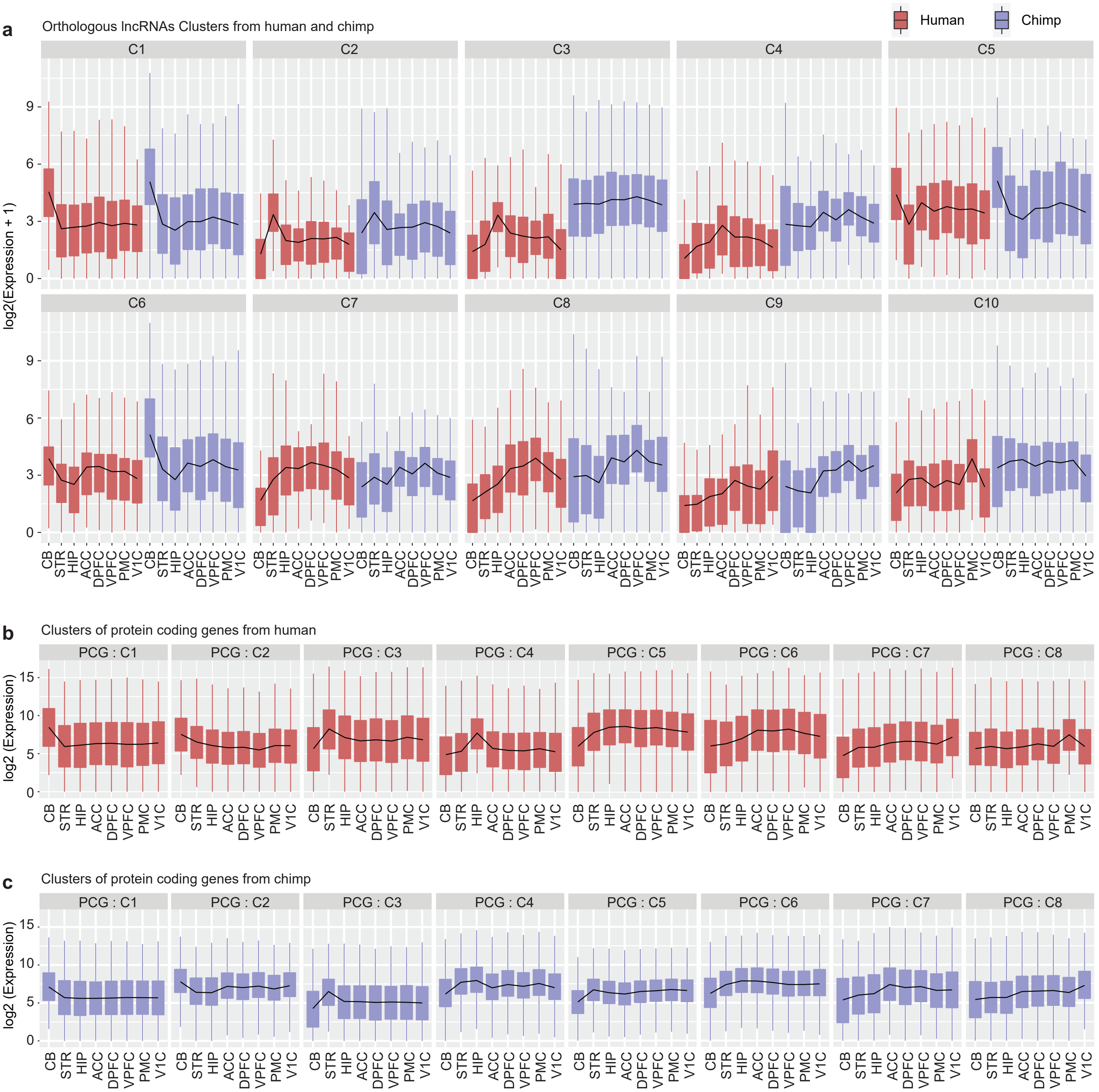

Supplement Figure 2:

(A) Boxplot representation for the clusters of expression profiles of DEO lncRNAs across different brain regions of the human and chimpanzee.

(B) Boxplot representation for the cluster of expression profiles of differentially expressed PCGs across different brain regions of the human.

(C) Boxplot representation for the cluster of expression profiles of differentially expressed PCGs across different brain regions of the chimpanzee.

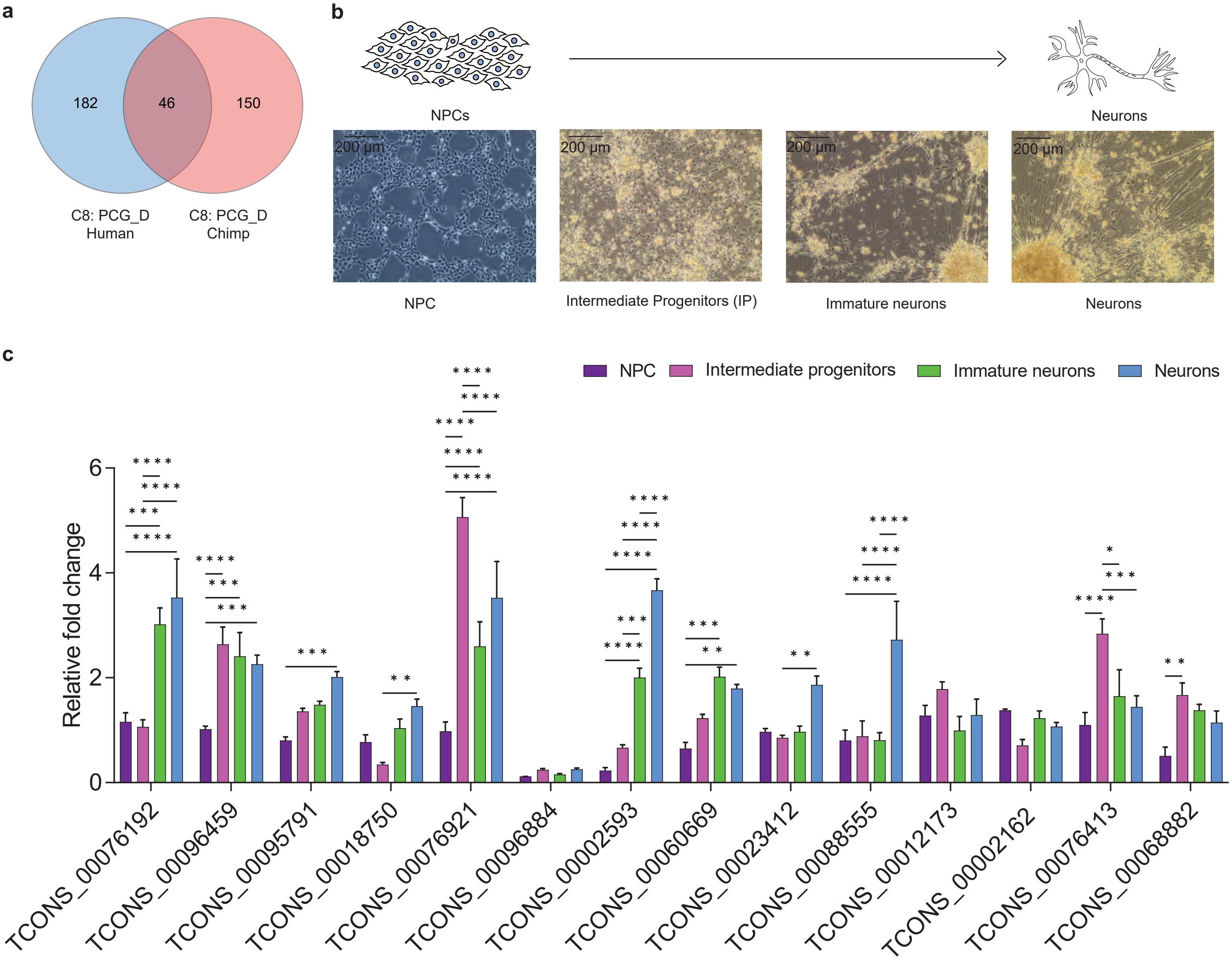

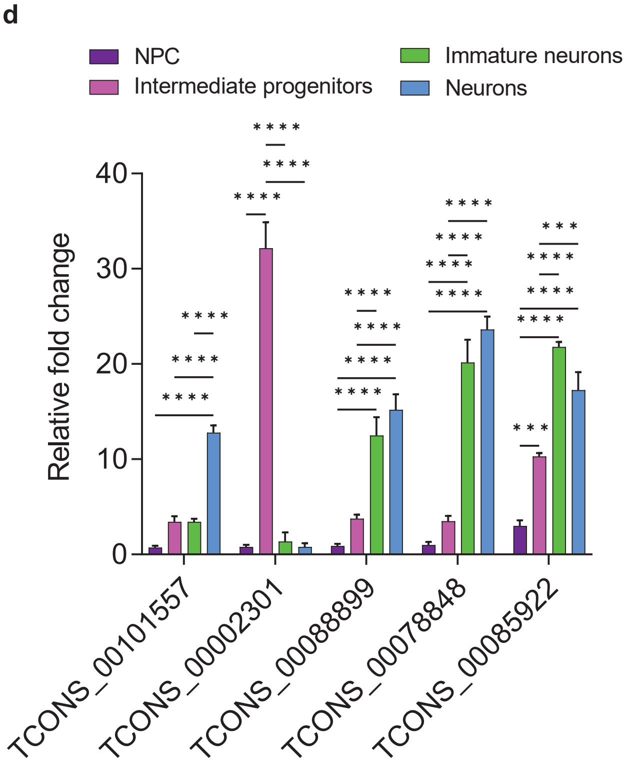

Supplement Figure 3:
(A) Venn diagram representing the total number of commonly and specifically regulated PCGs in C8 – PCG_D co-expression network of human and chimpanzee.

(B) In-vitro system used for validation of lncRNAs expression: Neuronal Progenitor Cells (NPC) → Neurons.

(C-D) Relative gene expression of the lncRNAs over the time of differentiation: neuronal progenitor cells (NPC), intermediate progenitor cells, immature neurons, mature neurons. 2-way ANOVA followed by Sidak's multiple comparison test. N=3 $*p<0.05$ $**p<0.01$ $***p<0.001$ $****p<0.0001$
