# Supplementary material for: Long non-coding RNAs expression and regulation across different brain regions in primates
Source: Sci Data. 2024 May 28;11:545. doi: 10.1038/s41597-024-03380-3 (PMC11133376; doi:10.1038/s41597-024-03380-3)
Supplement: Supplementary file 2 — Supplementary Files [file 41597_2024_3380_MOESM2_ESM.zip › Supplement_File5.docx]

**Supplement file: class code and of the gffcompare**

| **Class code** | **Meaning** |
| --- | --- |
| = | Complete, exact match of intron chain |
| c | contained in reference (intron compatible) |
| k | containment of reference (reverse containment) |
| m | retained intron(s), all intron matched or retained |
| n | retained intron(s), not all introns matched/covered |
| j | multi-exon with at least one junction match |
| e | single exon transfrag partially covering an intron, possible pre-mRNA fragment |
| o | other same strand overlap with reference exons |
| s | intron match on the opposite strand (likely a mapping error) |
| x | exonic overlap on the opposite strand (like 'o' or 'e' but on the opposite strand) |
| i | fully contained with a reference intron |
| y | contains a reference within its intron(s) |
| p | possible polymerase run-on (no actual overlap) |
| r | repeat (at least 50% bases soft-masked) |
| u | none of the above (unknown, intergenic) |
